# Validation of a Deep Learning System for the Full Automation of Bite and Meal Duration Analysis of Experimental Meal Videos

**DOI:** 10.3390/nu12010209

**Published:** 2020-01-13

**Authors:** Dimitrios Konstantinidis, Kosmas Dimitropoulos, Billy Langlet, Petros Daras, Ioannis Ioakimidis

**Affiliations:** 1 Visual Computing Lab, CERTH-ITI, 57001 Thessaloniki, Greece; dikonsta@iti.gr (D.K.); dimitrop@iti.gr (K.D.); daras@iti.gr (P.D.); 2 Innovative Use of Mobile Phones to Promote Physical Activity and Nutrition across the Lifespan (the IMPACT) Research Group, Department of Biosciences and Nutrition, Karolinska Institutet, 14152 Stockholm, Sweden; billy.langlet@ki.se

**Keywords:** eating behavior, meal analysis, meal duration, mouthfuls, bite-rate, eating patterns, deep learning, skeletal feature extraction

## Abstract

Eating behavior can have an important effect on, and be correlated with, obesity and eating disorders. Eating behavior is usually estimated through self-reporting measures, despite their limitations in reliability, based on ease of collection and analysis. A better and widely used alternative is the objective analysis of eating during meals based on human annotations of in-meal behavioral events (e.g., bites). However, this methodology is time-consuming and often affected by human error, limiting its scalability and cost-effectiveness for large-scale research. To remedy the latter, a novel “Rapid Automatic Bite Detection” (RABiD) algorithm that extracts and processes skeletal features from videos was trained in a video meal dataset (59 individuals; 85 meals; three different foods) to automatically measure meal duration and bites. In these settings, RABiD achieved near perfect agreement between algorithmic and human annotations (Cohen’s kappa κ = 0.894; F1-score: 0.948). Moreover, RABiD was used to analyze an independent eating behavior experiment (18 female participants; 45 meals; three different foods) and results showed excellent correlation between algorithmic and human annotations. The analyses revealed that, despite the changes in food (hash vs. meatballs), the total meal duration remained the same, while the number of bites were significantly reduced. Finally, a descriptive meal-progress analysis revealed that different types of food affect bite frequency, although overall bite patterns remain similar (the outcomes were the same for RABiD and manual). Subjects took bites more frequently at the beginning and the end of meals but were slower in-between. On a methodological level, RABiD offers a valid, fully automatic alternative to human meal-video annotations for the experimental analysis of human eating behavior, at a fraction of the cost and the required time, without any loss of information and data fidelity.

## 1. Introduction

The analysis of eating-related behavioral characteristics during meals is well-established in the field of microstructural analysis of human eating [1]. Such methodologies are mainly used in overweight and obesity research for understanding and modifying the behavioral mechanisms involved in long-term food intake (e.g., [2]). Similar interest exists in eating disorder research, where disturbed eating behavior has been associated with reduced long-term energy intake [3]. Other fields where detailed analysis of meal behaviors is important include disorder-specific eating behaviors [4], medicine-induced eating behavioral changes [5], dentistry research on temporomandibular disorders [6], the study of chewing and eating capacity in relation to aging [7], and even consumer research guiding the development of novel food products [8].

Traditionally, the estimation of meal behaviors has been based on self-rated measures, due to the ease and comparatively low cost of data collection, despite the significant increase on participant burden [9] and limited reliability, mostly due to erroneous reporting and reporting biases in many of the targeted populations [10]. Improving on these techniques, various methodologies for the objective quantification of eating behavior have been deployed, ranging from laboratory studies [11] to real life data collection actions [12]. The employed technologies cover a wide range of sensory modalities [13], with current advances supporting increasingly sophisticated data collection and analysis platforms. 

Despite those developments, the most widespread methodology for objective eating behavior analysis remains the analysis of meal videos, due to the advantages of the required data collection: (A) It reduces the participation burden. (B) It does not require additional wearable equipment. (C) It is appropriate for a range of conditions, from laboratory to pseudo-free-living settings. (D) It is becoming scalable even in free-living conditions, due to the increase in smartphone use. On the other hand, the large-scale meal video analysis also has disadvantages [14]: (i) Data collection is challenging without the participation of researchers; (ii) eating under observation might affect the study outcomes; and (iii) the analysis of the collected videos is time consuming and costly. Indeed, the manual annotation and analysis of meal videos require trained personnel and significant time, with the process often lasting significantly more than the data collection itself. Thus, methodological advancements improving behavioral analyses of meal video recordings are often requested by researchers [15], in order to standardize and optimize the required process. 

The duration of meals is one of the main behavioral parameters that is routinely studied in such settings, usually in parallel with total meal food intake [16]. Indeed, current evidence associates the speed of eating with overweight and obesity, with increased speed of eating contributing to higher energy intake [17], potentially being associated with the development [18] and maintenance of obesity [19]. Another widely studied behavioral parameter is the number of bites individuals take during meals, which has been previously associated with the portion size of a meal [2,20], with some researchers proposing the quantification of bites as an alternative for energy intake measurements [21]. Overall, the behavioral analysis of meals with different foods has revealed that most individuals maintain their eating profile in laboratory conditions [22], allowing meal characteristics to be used to classify individuals [16]. Regarding the transferability of such measures beyond controlled settings, real-life studies are certainly needed [23], but evidence shows that behavioral measures in semi-controlled settings [24] can carry over to free-living settings [25]. 

Finally, the longitudinal analysis of meal progression previously led to the identification of meal progress patterns (i.e., decelerated vs. linear eaters [26]) associated with eating disorders and obesity [27]. However, such analyses are seldom, due to their inherent complexity and a lack of appropriately annotated datasets to support them.

Our current effort focuses on the development of a tool that improves the already existing and well-established video meal analysis methodology that can be used for the automation of the behavioral meal annotation process, on the level of meal duration and meal bites. Our proposed methodology is supported by recent advances in the fields of deep learning and image processing for skeletal feature extraction that led to the detection and analysis of specific movements in videos [28] and has been developed retrospectively on top of existing video meal datasets collected in controlled settings. Thus, we designed and deployed the “Rapid Automatic Bite Detection system” (RABiD), attempting to eliminate the need of human annotation for meal duration and total meal bites. We aimed at achieving this without any information and fidelity loss, outputting behavioral information appropriate for group-level and within individual analytics. Moreover, we are proposing a novel method for meal-progress biting rate analysis that can be useful for more detailed behavioral meal analysis.

The remainder of the paper is as follows: Section 2 presents the experimental setup, the training of RABiD algorithm, the selected validation methodology, and the statistical methods used for the analysis of results. Section 3 presents the experimental results of the validation of RABiD and the comparison with the manual human annotation procedure. Finally, Section 4 discusses the findings of this study, while Section 5 concludes the work.

## 2. Materials and Methods 

### 2.1. Experimental Design

**Figure 1:**
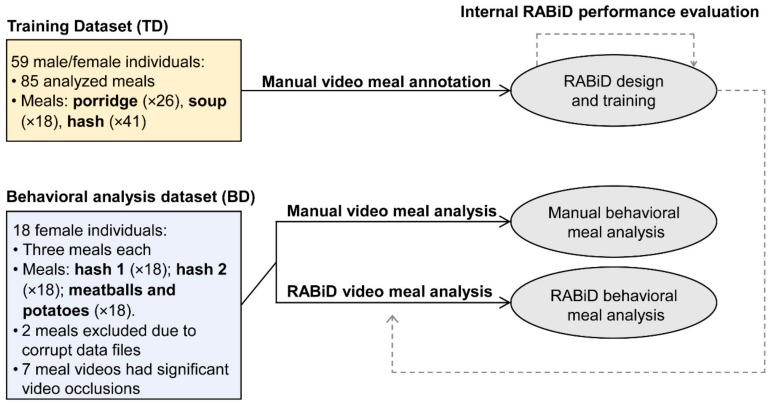
Schematic representation of the experimental design. The training dataset was used for the design, training, and internal performance evaluation of Rapid Automatic Bite Detection (RABiD). RABiD was later used to analyze the behavioral analysis dataset and the outcomes of this analysis were compared with the analysis outcomes based on manual video annotations.

Two separate datasets of meal video recordings (Figure 1) were used for this study. RABiD was initially trained on an independent training dataset (TD) of meal videos recorded from individuals consuming three different food types under identical controlled conditions. These meals were independently annotated by one trained human annotator, as the basis for RABiD’s creation/training. Afterwards, a separate dataset of meal video recordings (behavioral analysis dataset; BD) was analyzed and the behavioral outcomes of manual human annotation analysis were compared with those of RABiD. In the TD, the majority (66%) of participants consumed only one food type, while the rest (44%) consumed two different meals. However, in the BD, all subjects participated in all three meals. Both datasets were collected earlier (2013–2015) and were later used for the development and evaluation of the RABiD methodology (September 2019). In all the presented protocols, the data were collected in accordance with the guidelines for human research in the Declaration of Helsinki and were approved by the Stockholm Regional Ethics Board (Dnr. 2012/219-31/5, 2014/535-31/3 and 2015/2003-31). The analyzed datasets include a mix of control measurements presented in [16], complemented with additional, not previously analyzed, meals.

### 2.2. Recruitment

The recruitment process was identical both for the TD and BD meals. Overall, our studies focused on the analysis of eating behavior in healthy individuals, with inclusion criteria being non-vegetarian young adults (18–35 years old) with close-to-normal body weight (body mass index (BMI): 18–27 kg/m^2^). Since the BD focused on individual responses inside a homogenous group of participants, only females were recruited for this study. The participants were recruited online and through poster advertisements. Respondents attended an initial information meeting covering the protocol of the study and they agreed in participation by signing an informed consent. Afterwards, the participant’s weight and height were measured and information about their general health status was collected. For these studies, pregnant women, as well as individuals with past diagnoses of eating disorders were excluded. Similarly, we excluded individuals who had food-specific allergies or declared a strong dislike for the experimental foods. Participants were aware that their meals were video recorded and analyzed but were not aware of the specific outcome parameters (such as meal duration and bite analyses). All the subjects were rewarded with one cinema ticket per meal for their participation. 

### 2.3. Meal Session Procedure

All meals were provided to participants during typical Swedish lunch hours (11 a.m. to 13 p.m.) and participants were instructed to abstain from vigorous physical activity before the meal sessions. Additionally, the subjects were instructed to eat breakfasts at least 3 h prior to the recorded meal sessions. In cases of repeated experimental meals (i.e., a subset of the TD and everyone in the BD), the experimental sessions were scheduled at least a week apart and participants were asked to consume similar breakfasts during experimental days (as reminded by messages on mobile phone the day before). All meals took place in dedicated experimental rooms without windows where the subjects ate alone, without access to other activities (e.g., listening to music, reading or using mobile phones). Foods were presented in trays (or bowls in the case of soup and porridge in the TD) on the dining table, in appropriate quantities in order to create a sense of ad libitum food availability. The participants were informed that more food (on top of the amount presented on the table) was available, if desired, and that they could eat as much as they wanted. No time limit was set for each meal, allowing subjects to finish their meals at their own pace. The participants transferred food from the serving trays/bowls to their own plate/eating bowl, placed directly in front of them, as many times as they wished. Water in large glasses was served together with the food, without any restraints about frequency, volume, and timing of drinking. After the initial instructions, the researcher exited the dining room and returned only after the termination of the meal. This procedure, as well as the recording location, was identical for TD and BD meals, and similar to practices that we previously published [3,16,29].

### 2.4. Served Foods

**Figure 2:**
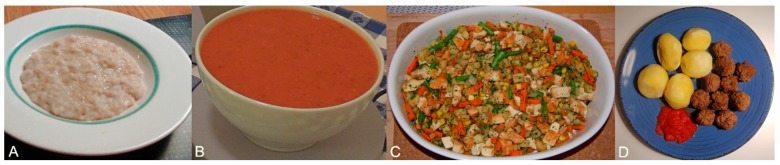
The foods served in the meals analyzed here: (**A**) porridge (served in the TD), (**B**) soup (served in TD), (**C**) hash meal (served in TD and BD), (**D**) meatballs and potatoes (served in BD).

Three different food types were served for the meals included in the TD and one additional for the BD (Figure 2). In short, for the TD we served porridge, soup, and hash meals. Similarly, in the BD we served an identical hash meal twice (hash 1 and hash 2 in Figure 1) and a meatballs and potatoes meal once, in order to facilitate the planned within-subject analysis. Thus, the two selected foods were significantly different in food unit size and nutritional characteristics, in order to test the ability of RABiD to reliably detect the (expected) behavioral differences due to differing food properties. The nutritional characteristics of the served foods can be seen in Table 1 and additional information about the food characteristics, preparation, and cooking can be found in the Supplementary Material (Methods S1).

### 2.5. Video Capturing and Data Handling

**Figure 3:**
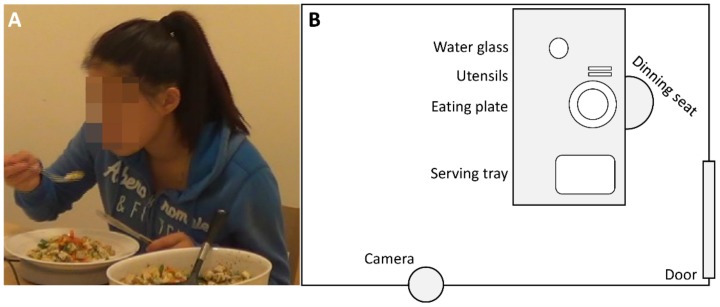
(**A**) Screenshot from an experimental meal video. (**B**) A schematic representation of the experimental dining room. The subjects were free to change the placement of the items on the table at will. There were no other restrictions for movements and positioning during the meal.

Since all the videos analyzed in this study were originally recorded to support manual behavioral annotations only, no special attention was given to factors that would potentially affect the performance of an automatic annotation algorithm, like the angle of recording, the occlusion of the subject’s skeleton, and the lighting of the room. Thus, all the meals were videotaped using one digital camcorder (Samsung, Suwon, Korea) that was placed on the left front side of the subject eating, at an angle of 40°–45°, at a distance of approximately 1.5 m from the eating position. The videos were recorded at 576 p resolution (720 × 576 pixels), at 25 frames per second. The camera was mounted on a shelf using a “mini-gorilla” stand at a height of ~1.2 m. The camera positioning was maintained stable across meal recordings, and the lighting of the room was never modified (i.e., typical soft white light from incandescent bulbs placed on the ceiling of the room). Additionally, the subjects did not receive any directions concerning their eating position, body stance, or movements during the meal. Similarly, the placement of the serving trays/bowls and the water glasses was not restricted (Figure 3). The camera was turned on before the entrance of the subject into the experimental room and turned off after their exit. The video files were saved on flash memory cards in the camcorder and were later transferred to PCs for further analysis. At this stage, two videos were excluded due to corrupt video files. 

### 2.6. Manual Video Annotation

The meal videos were annotated using The Observer^®^ XT v12.5 (Noldus Information Technology, Wageningen, Netherlands) with the human annotator marking five behavioral events: (a) “meal-start”, the timing of the initial “spoonful”; (b) “meal stop”, the timing of the last “bite”; (c) “spoonful”, the moment that food leaves the personal eating plate (only if the food eventually reaches the mouth of the individual, without the utensil returning to the plate; (d) “food addition”, the act of transferring food from the serving tray into the personal eating plate; and (e) “bite”, the moment that food enters the mouth of the individual. Plotting these events in time resulted in a timestamped behavioral log for each meal. All of the analyzed meals were manually annotated by the same trained researcher, minimizing the potential of intra-annotator errors in the behavioral meal logs. The human researcher performed this task while the video file was played in half speed, in order to achieve more accurate annotations. Prior to the analysis of the BD, with the help of RABiD, another human annotator watched all the BD videos and rated the video stream for visible occlusions of the mouth and hands of the participants (not visible from the camera). The annotator evaluated potential occlusions due to sitting position (e.g., individuals using their right hand for bites while facing away from the camera, resulting in limited view of the “eating hand”) and placement of items on the table (e.g., serving tray placed too close to the eating plate hiding it from the camera view). The videos were ranked with regards to occlusion as: “minimal” (33 videos), “medium” (12 videos), and “significant” (7 videos). 

### 2.7. Data Preparation for Automatic Behavioral Meal Analysis

There was no special preparation of the video files for the design and the deployment of RABiD in the TD and BD. For the TD, the annotated behavioral logs, in full detail, were used for the training RABiD. For the BD, the only information available before the RABiD analysis was the first and last points when the research subject was alone in the room (i.e., when the researcher exited and re-entered the room before and after the meal). Thus, no specific information for the actual meal duration and timing of the bites was used, blinding the RABiD analyses of the BD. 

### 2.8. Algorithm Training

**Figure 4:**
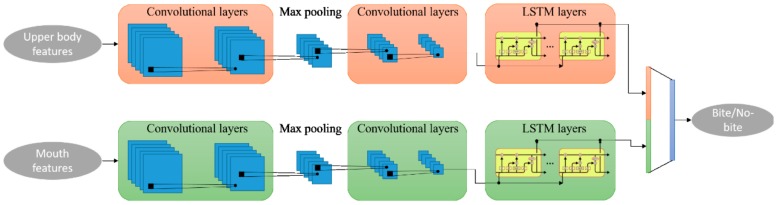
Schematic representation of the RABiD algorithm, for the parallel analysis of upper body (including the head) and mouth movements in videotaped meals. The outcome of the algorithm is a complete behavioral description of a single meal including meal duration, total number of meal bites, as well as time series of bite instances across the meal. LSTM: called long short-term memory.

**Figure 5:**
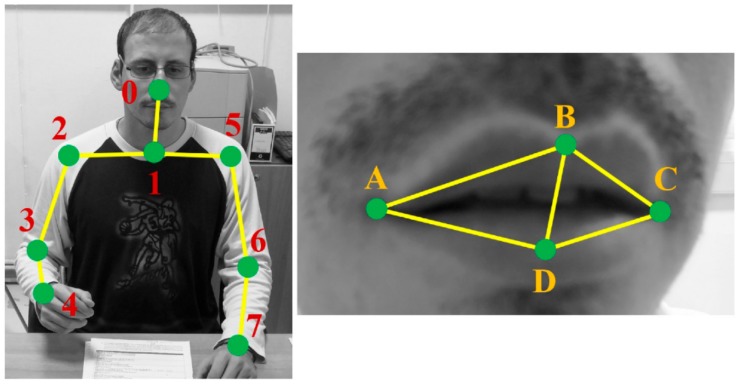
Schematic representation on a researcher of the skeletal joint (0–7) and mouth (A–D) points tracked and analyzed in the RABiD algorithm. Both two-dimensional (2D) coordinates and distances are analyzed over the meal progression.

RABiD is a deep learning-based algorithm for the processing of videos and classification of bite instances. It consists of two data streams that receive upper body and mouth features, respectively (Figure 4). Specifically, the first stream employs the two-dimensional (2D) coordinates of the nose and hand skeletal joints and the distances between them (i.e., 0–7 numbered skeletal joints in Figure 5), while the second stream employs the 2D coordinates and distances of three mouth points that correspond to the middle of the upper and lower lips and the corner of mouth (i.e., B, D and A or C points based on which side of the user is visible while eating in Figure 5). The algorithm was implemented in Python, while the Keras-Tensorflow frontend-backend was also used for deep learning.

RABiD was designed based on the supposition that the movement of hands and the mouth are the most significant motor indicators of bite instances. RABiD applies the same processing procedure in the two streams’ input of upper body and mouth features. It initially extracts more discriminative spatiotemporal information using two blocks of convolutional layers that are responsible for computing interactions between neighboring features both in time and space. The max pooling operation between the convolutional layers downsamples the feature space and improves the robustness of RABiD. Afterwards, a series of recurrent neural network components, called long short-term memory (LSTM) units are employed to extract new temporal information from the highly discriminative feature sequences computed from the convolutional layers by learning long-term dependencies in the feature sequences. Finally, the computed features from the two streams are concatenated and fused together, using a fully connected layer that combines the information from the hands, head, and mouth to achieve more accurate and robust bite detection results.

The TD meal videos were cropped into 12,121 video clips with a duration of 2 s and formed an isolated dataset with 4149 bite instances and 7972 non-bite instances, based on the manual annotations of the dataset. Initially, 90% of these video clips were used for training RABiD. For the extraction of hand and mouth features, we used OpenPose, a previously developed deep learning algorithm [30,31] that extracts skeletal features from images. The current version of the analysis processed videos for the extraction of skeletal features in a much higher resolution than before [27], producing more accurate hand and mouth features. The outcome measures are then processed to remove abnormal values (i.e., outliers) or to fill out missing values (i.e., non-detected joints) with values of the previous time frame containing a value for the specific feature. Finally, the movement is smoothed using spline interpolation and the coordinates are normalized by transformation into a local coordinate system with the neck and nose as origins for the upper body and mouth features, respectively. The last processing step aims to diminish the influence of the location of a person in the video, as well as to provide smoother skeletal joint/mouth point movements.

The remaining 10% of the clips (i.e., 1212 clips containing both bite and not-bite instances, not used for the training of the algorithm) were used for performing internal (i.e., inside the TD dataset) evaluation of the algorithm performance for iterative optimization of the RABiD parameters, until sufficient accuracy was achieved. 

### 2.9. Automatic Meal Analysis

During the automatic meal analysis, an entire meal video is fed as input to RABiD which is used to detect the precise time frames during which bite instances occur. This is achieved by employing an overlapping window of 2 s with a step of 1 frame, thus computing a bite probability for each frame of the videos. The final output of RABiD is a continuous signal of bite detection probabilities. This signal is then post-processed for the extraction of the exact time frames that correspond to true bite instances and for the removal of false detections. This is achieved by using medial filter smoothing for removing small and abrupt changes in the bite probability signal, as well as using the sum of the mean and standard deviation of the bite probability as a threshold for removing false bite detections. The post processing process is completed with the computation of local maxima and the removal of maxima that are too close to each other. The remaining local maxima corresponds to the true bite instances detected by RABiD.

### 2.10. Statistical Analysis of Behavioral Outcomes

Initially, for the internal TD evaluation of RABiD, we calculated Cohen’s kappa (κ) to measure the agreement among the human annotation and the RABiD outcome, on the level of “bite” and “not bite” labeling of a video clip. The κ ranges from −1 to +1, with values κ ≤0 indicating lack of agreement between human manual and RABiD annotations, whereas κ-value ranges between 0.60 and 0.80 indicate satisfactory agreement and κ >0.80 pointing to very high (near perfect) agreement [32]. 

After the completion of the automatic analysis of the BD, the behavioral meal logs produced by RABiD were compared with the behavioral meal logs produced by the independent manual annotation of the meals. In the subsequent analyses, these datasets are referred to as manual and RABiD. All the presented analyses were performed twice, once for the whole BD (52 meals) and once with the exclusion of seven meals ranked as having significant occlusions (45 meals). Here, since the overall results were very similar, we present and comment on the latter analyses.

For comparisons across the meals of the same individual (hash 1 vs. hash 2 vs. meatballs) we used a linear mixed effects model using the lme function of the nlme package in R 3.2.3. Condition-specific comparisons were performed by Tukey post-hoc tests, using untransformed values, setting the condition as the fixed effect and the subjects as the fixed effect, with random intercept and fixed slope. Shapiro–Wilk tests and visual inspection of Q-Q plots and residuals vs. fitted value were used to test the assumption of normality for the presented measurements. The agreement between total bites per meal and meal duration (manual vs. RABiD) were evaluated through Pearson correlations, with “medium”, “high”, and “very high” thresholds set at R2 ≥0.50, 0.75, and 0.90, respectively [33]. 

Finally, for the microstructural longitudinal analysis of bite rate progression as a meal is progressing, we followed identical post-processing both for the manual and RABiD bite time series. Initially, the bite occurrences per 10% of meal segment were calculated for all meals. Then an average value for each time segment of an individuals’ hash meals 1 and 2 was calculated. Finally, the average rate of change of bite rates as a meal progressed was modeled through a quadratic curve fit (Sigmaplot 12.5, Systat Software, San Jose, CA, USA) for each meal type (hash vs. meatballs). In this case we did not perform additional statistical testing between meal types, or manual vs. RABiD outcomes, due to lack of statistical power resulting from multiple comparisons. Thus, we are presenting a descriptive visual analysis of the resulting curves. The significance threshold of all the performed statistical tests was set at 0.05 and all the values presented in the text are mean (SD), unless otherwise specified. 

## 3. Results

### 3.1. Subjects

A total of 77 subjects contributed data for the study (Figure 1). Out of those, the meals of 59 males and females were included in the TD and the meals of 18 different females comprised the BD. The characteristics of the subject samples for the TD and BD meals can be found below (Table 2).

### 3.2. Internal RABiD Algorithm Performance Evaluation in the Training Dataset

The level of agreement between manual and RABiD annotations, based on the isolated subsample of video clips from the TD, consisting of 1212 samples (10% of total), was near perfect (κ = 0.894). The detailed results of the comparison of the RABiD outcomes vs. the manual annotations are presented in Table 3, where each cell corresponds to the agreement/disagreement of results for manual vs. RABiD video clip annotation (e.g., 374 out of 1212 video clips were identified as containing a bite both from the manual and RABiD annotations).

### 3.3. Meal Duration and Total Meal Bites in the Behavioral Dataset

The analysis of the manual annotations revealed that individuals took significantly fewer bites (*p* < 0.01) during the meatball meal in comparison to hash 1 meal. There were no significant differences in the total number of bites between the two hash meals and no differences in meal duration either between hash 1 vs. meatballs, or hash 1 vs. hash 2 meals (*p* > 0.05 in all cases). The results were identical when the same analysis was performed based on the outcomes of the RABiD algorithm (*p* < 0.01 for hash 1 vs. meatballs and *p* > 0.05 for all other comparisons). Table 4 presents these results in detail.

### 3.4. Manual vs. RABiD Meal Duration and Total Meal Bites Correlations

**Figure 6:**
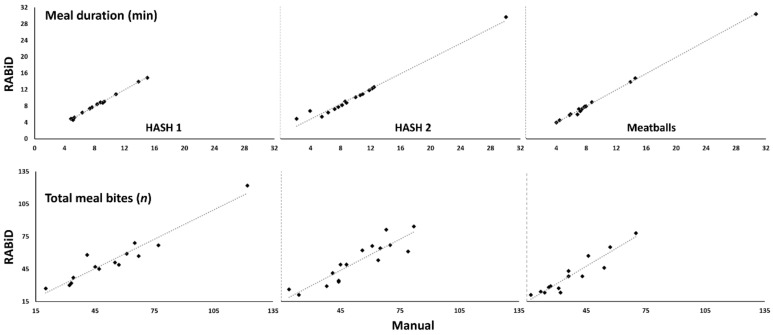
Scatter plots of the meal durations and total meal bites for all the analyzed meals in the behavioral dataset. Very high agreement is observed between manual and RABiD measures in all cases.

The overall correlations between the manual and RABiD measures of meal durations were 1.00, 0.99, and 1.00 for hash 1, hash 2, and meatball meals, respectively, showing very high agreement between manual, even when individual meals are considered (total correlation coefficient for meal duration was 0.99). These results are presented in Figure 6.

Similarly (Figure 6), when considering the annotated (manual) vs. detected (RABiD) total meal bites, the correlations were also very high, with coefficients of 0.96, 0.91, and 0.94 for the hash 1, hash 2, and meatball meals, respectively. As before, these results point towards very high agreement of annotated vs. detected total meal bites, even on an individual level (total correlation coefficient for number of bites is 0.94).

### 3.5. Meal Progress Analysis

**Figure 7:**
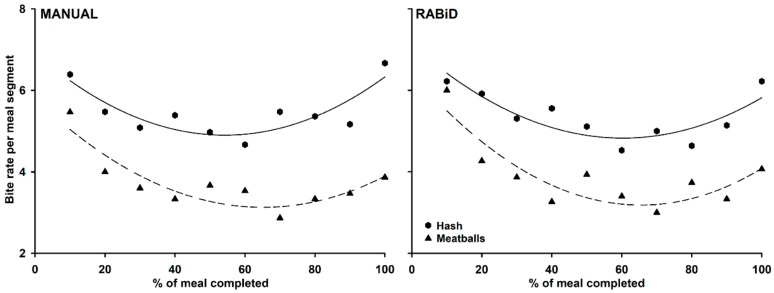
Changes in bite rates during hash and meatball meals, as revealed by manual annotations and RABiD analysis outcomes. Bite rates per meal segment are calculated as bites during 10% of meal duration (bites/s).

Figure 7 depicts the changes in bite rate as a meal progresses (in 10% time segments), both for the manual and RABiD analyses (function: $y_{0}+ax+bx^{2}$; hash coefficients: y_0_ = 6.909, a = −0.7409, b = 0.0683 for manual and y_0_ = 7.116, a = −0.7590, b = 0.0629 for RABiD; meatball coefficients: y_0_ = 5.797, a = −0.8189, b = 0.0629 for manual and y_0_ = 6.407, a = −0.9849, b = 0.0753 for RABiD; *p* < 0.05 for all model fits). Overall, the fitted quadratic curves reveal a similar pattern for hash and meatball meals, where individuals take bites more frequently at the beginning and end of the meals, with slower biting rates in between.

### 3.6. Time Efficiency of the Analyses

The precise time efficiency of the manual annotations of meals is a challenging measure to calculate, since it is significantly influenced by many uncontrolled factors, including behavioral annotation setup, annotator experience, rate of annotation errors and correction, complexity of annotated behavior, and experimental subject behavioral profile. Probably the most important factor for the complete annotation of such an extensive dataset of meal videos is the annotator fatigue and time availability. Here we are estimating that the annotation of each video took approximately twice as long as the duration of the meal (due to half-speed playback during annotations). We are also adding 35% on this figure accounting for annotation setup, file management, and human error corrections. Based on the above suppositions, the total duration for the manual annotation of the presented datasets was close to 44.4 h for videos in the TD and 24.6 h for those in the BD. Obviously though, this effort was scattered across a significantly broader time period, dependent on personnel availability and work load (close to seven calendar months in our case).

In contrast to manual analysis, the time efficiency of RABiD can be computed with more precision. Specifically, based on the available hardware (medium performance level Windows workstation with a NVidia GTX980 video card), the extraction of the skeletal characteristics in OpenPose and the subsequent processing steps lasted, on average, 37.7 (17.0) min per video. This calculation includes both TD (55.1 h) and BD (34.5 h) videos, since the OpenPose component is required in all cases. Additionally, training of the RABiD algorithm was performed only once and lasted 2.4 h. After the OpenPose analysis was completed the trained RABiD algorithm was able to detect bite instances on any video that was given as input. It should be noted that all the estimations above are heavily dependent on the selected analysis resolution, the available hardware, and the duration of the analyzed videos. While this is certainly a resource-heavy analysis, it should be also noted that it was completely automatic and, for the presented dataset, all the steps were completed in approximately 10 consecutive calendar days. This time period also accounts for intermediate data-control procedures, with the final data retention rate being 100% (i.e., no data were corrupted or lost).

## 4. Discussion

This study evaluates a new methodology for the automatic behavioral analysis of meals on the level of meal duration and bites, based on video meal data recorded in controlled environments. Self-rated methodologies, which are predominantly used for estimating eating behavior, being cost effective and easy to analyze, rely heavily on the participant’s input, but often suffer from reliability issues [10]. On the other hand, emerging objective methodologies for automatic meal analyses [29] are often based on wearable technologies, which can affect the participants’ behavior, or on proprietary additional equipment [34], affecting the scalability and the cost-effectiveness of the performed studies. Efforts to use off-the-shelf wearable technologies (e.g., smartwatches) for meal analyses are promising [35], but they are currently not widely available for large-scale use. Thus, the predominant methodology for objective, non-invasive behavioral analysis of meals is the use of video observation, especially for studies performed in controlled [16] and semi-controlled settings [36]. Further, video meal analysis is usually the “ground truth” for the development of other objective analysis methodologies. However, this methodology is still limited [15] by its dependency on time-consuming and error-prone manual video annotations, with many studies resorting to the use of multiple human annotators, expertly trained to achieve increased reliability. 

In order to eliminate these shortcomings, we developed and trained RABiD using a training set of meal videos, including three foods with significantly different textures. Using common algorithm performance metrics, we initially performed an internal algorithm outcome validation, using a random fraction (10%) of the video clips that were produced during the post-processing of the training meals. Then we evaluated RABiD against traditionally performed analyses based on manual human annotations on an independent set of meal videos, collected in the past, as part of our usual eating behavioral research.

Regarding the internal RABiD outcome validation, we managed to increase our previously published performance [28], using higher resolution images for the extraction of skeletal features. These features were then used for even more accurate modeling of hand, head, and mouth movements on a 2D plane. While our previous results already achieved a near-perfect agreement with manual video-clip annotations (κ = 0.879) [28], our current results improve the rate of agreement (current κ = 0.894), pointing to superb algorithm performance. On that level, RABiD outperforms all previous comparable efforts of video-based and wrist-worn accelerometer meal analyses. Earlier video-based methods employed spectral segmentation, random forest classification [37], and hidden Markov models in order to quantify dependencies between hand gestures and bite instances [38], with satisfactory agreement (0.60 < κ < 0.80) on bite detection results. More recent bite detection systems, using wrist-worn sensors, deploy deep learning methodologies [35,39] in order to propose more accurate and robust solutions and produce promising results. Such research creates optimism about the future, showing that parallel advancements in video-based and wrist-worn sensor solutions are en route to significant advancements in the field of automated meal analysis. 

While the internal validation of RABiD performance is important, a more real-life validation test for the algorithm is its performance on independent meal videos is needed, as compared to the same analysis performed by human annotators. When these analyses were compared against each other (RABiD vs. manual), the outcomes were interchangeable (i.e., both methodologies detected similar group effects; Table 4). Thus, both analyses pointed towards group maintenance of average meal duration (8.4 vs. 8.3 min in hash 1 and 9.8 vs. 10.2 in hash 2 for manual vs. RABiD, respectively) and mean total bites per meal (54 vs. 53.6 in hash 1 and 53 vs. 51.3 bites in hash 2 for manual vs. RABiD, respectively) when the same food was consumed twice by the same individuals. When the same individuals consumed a different food, both the manual and RABiD analyses detected significant decreases on the mean total number of bites per meal (37.1 vs. 38.9 bites for manual vs. RABiD, respectively), while the mean meal duration remained similar (9.3 for both manual and RABiD). Similarly, the RABiD performance was good enough even for performance of within subject analyses, as revealed by the very high nested correlation coefficients (Figure 6) for each type of recorded meal, regarding both: (i) the quantification of meal duration (the lowest correlation coefficient was 0.99 for hash 2) and (ii) the quantification of total meal bites (the lowest coefficient was 0.91 for hash 2). In effect, using RABiD exclusively in the absence of any human behavioral meal annotation, would produce equally valid behavioral outcomes as traditional analysis technics.

Regarding the behavioral outcomes of the present study (irrespective of using the RABiD or manual methodologies, since they are similar), it should be noted that they are directly comparable, but not identical, with our previously published results. Specifically, when subjects in the BD consumed the same food served in identical settings (hash 1 vs. hash 2), no significant changes in meal duration and the total meal bites were observed. This supports our [16] (and others [22]) argument that in a stable setting, cumulative eating behavior remains constant, both on a group and an individual level. On the other hand, the introduction of a food with larger food units resulted in a significant drop of total meal bites, but did not affect the total meal duration, resulting in equally long meals, with individuals taking fewer, but probably bigger, bites. These results support our past argument that food type is an important modulator of eating behavior across a meal, despite fairly constant individual eating styles [16]. It should be emphasized that our present experimental design did not control for food differences on the level of texture, taste or nutritional characteristics. However, we remain hopeful that the introduction of RABiD-like methodologies will make the performance of additional studies easier, facilitating the proper evaluation of the behavioral effects of such parameters.

Finally, when we modeled the rate of change of the bite rate throughout the meal progression, the results, again, appear identical for the manual vs. the RABiD analyses. We specifically observed higher biting rates at the beginning and end of the meals, with lower biting rates during the meal mid-point. This pattern repeated for both foods (mash and meatballs), despite the fact that the absolute recorded rates were different (overall higher for hash vs. meatballs, Figure 7). The specific identified pattern is moderately unexpected, since it does not agree with previously described food intake curves that point either to a steady or a decelerated eating rate across meals [26]. This discrepancy reveals that biting rate should not be regarded as equivalent with food intake rate as proposed before [21]. The observed differences may point towards the fact that meals are initiated with larger and more frequent bites, are followed by less frequent bites, and are concluded with frequent, but smaller, bites. The emergence of more powerful analysis techniques, such as RABiD, will facilitate more detailed meal-progress behavioral analytics producing more information about how different groups of individuals (e.g., lean vs. obese groups) consume their foods.

Initially, it should be noted that the RABiD methodology was developed exclusively for the analysis of meal videos recorded in controlled or semi-controlled environments. The selected deep learning algorithm module (i.e., OpenPose [30,31]) used for the extraction of the skeletal and mouth characteristics is pre-existing and fairly computational-heavy, making its deployment and its time efficiency dependent on the available hardware. However, we are foreseeing that further optimizations of the deep learning module can maintain the performance of the algorithm, while significantly improving its time efficiency. Also, even at the current time efficiency levels, the automatic nature of the analysis allows it to be completed at a fraction of the time that would be required by a human annotator (10 days vs. 7 months of real-time analysis period). Additionally, current hardware developments (e.g., supporting Artificial Intelligence acceleration on dedicated mobile phone processors [40]) are expected to allow such analyses to be performed even on videos captured by smartphone cameras, potentially on a close to real-time speed, making them scalable and relevant even for real-life. Similarly, it should be noted that in this effort, RABiD was trained and evaluated using meal videos captured at an angle (40°–45°). This was necessitated by the availability of existing meal videos and it proves the usefulness of RABiD for such retrospective analyses. However, the use of side-viewing videos affects the performance of the algorithm, mostly due to potential occlusions of the skeleton of the subjects. Thus, the next logical step would be the training of similar algorithms on frontal meal recordings, in order to achieve improved algorithm performance. Finally, the current effort concentrated on the analysis of eating plated food with utensils and does not necessarily translate well to the automatic analysis of eating hand-held food items (e.g., sandwiches, burgers, etc.). In such cases, the successful automatic annotation of behaviors should also work following an identical methodological approach, but training another instance of RABiD, dedicated to hand-held foods, might be required in the future.

Concluding, the present study, together with parallel efforts in this area, reveal that automatic analysis of meals, using wearables, video or other sensory equipment, is gaining ground, especially in applications related to personal nutrition [41]. Indeed, the domain is rapidly reaching the point when the need for manual human annotations will not be required for the detection and the analysis of food intake and the related behavioral characteristics, allowing researchers to optimize their data collection and data analysis methodologies.

## 5. Conclusions

In this work, we propose and analyze an automatic food bite detection algorithm, called RABiD, that can eliminate the need for human annotations of video-based meal analysis. In short, our results support the notion that fully automated deep-learning-driven methodologies can support behavioral analyses of meal duration and meal bites as well as those based on human annotators. More specifically, based on the outcomes of our study, we argue that we have managed to achieve a detailed behavioral analysis of meals without any loss of information and fidelity, in a fraction of the time (and effort) that would be required if the meal annotation was performed by trained human annotators.

## Figures and Tables

**Table 1:** Nutritional characteristics of the foods served in the meals of the TD and BD.

	Porridge	Soup	Hash	Meatballs and Potatoes
Served (dataset, *n* of servings)	TD, ×1	TD, ×1	TD, ×1/BD, ×2	BD, ×1
Protein (/100 g)	4.2	2.1	9.6	9.4
Carbohydrate (/100 g)	20.9	5.9	8.2	8.5
Fat (/100 g)	2.1	4.6	2.0	6.8
Energy (kJ/100 g)	496.4	327.6	383.3	585.6

**Table 2:** Group characteristics for the two assembled datasets.

	Training Dataset (*n* = 59)	Behavioral Analysis Dataset (*n* = 18)
Males/Females	21/38	-/18
Age, years	26.2 (5.1)	25.9 (4.7)
Height, cm	168.6 (8.8)	164.1 (5.4)
Weight, kg	64.9 (10.2)	60.6 (7.7)
Body Mass Index, kg/m^2^	22.7 (2.1)	22.5 (2.1)

Values are expressed as mean (SD).

**Table 3:** Confusion matrix, recall, specificity, and F1-score of the RABiD algorithm performance in the evaluation subsample of video clips in the TD (*n* = 1212).

	Clips Including Bites (RABiD)	Clips Not Including Bites (RABiD)
Clips including bites (manual)	374	27
Clips not including bites (manual)	30	781
	Recall: 0.933; Specificity: 0.963; F1-score: 0.948	

**Table 4:** Meal duration and total number of bites for three repeated meals from the same individuals; * *p*-value <0.05 from linear mixed model comparing hash 1 vs. meatball meals.

		Hash 1	Hash 2	Meatballs
**Manual Analysis**	Meal duration (min)	8.4 (3.2)	9.8 (6.2)	9.3 (6.6)
	Total meal bites (*n*)	54.0 (25.2)	53.0 (18.2)	37.1 (14.6) *
**RABiD Analysis**	Meal duration (min)	8.3 (3.2)	10.2 (5.7)	9.3 (6.6)
	Total meal bites (*n*)	53.6 (23.7)	51.3 (19.3)	38.9 (17.0) *

## Supplementary Materials

The following are available online at https://www.mdpi.com/2072-6643/12/1/209/s1, Methods S1: Food characteristics, preparation and cooking.
