# Supplementary material for: Validation of a Deep Learning System for the Full Automation of Bite and Meal Duration Analysis of Experimental Meal Videos
Source: Nutrients. 2020 Jan 13;12(1):209. doi: 10.3390/nu12010209 (PMC7020058; doi:10.3390/nu12010209)
Supplement: Supplementary file 1 [file nutrients-12-00209-s001.pdf]

Supplementary Material

Food characteristics, preparation and cooking

In the **Porridge meal**, the subjects were served oat-based porridge prepared with oats-flakes (ICA AB, Sweden), milk (fat content 1.5%), water, salt and sugar, and cooked in boiling water for ten minutes. In the **Soup meal**, the participants were served a pre-cooked tomato soup (ICA AB, Sweden), finely grinded together with pre-cooked blended pork and beef minced meat (20% fat) and salt (ICA AB, Sweden). Finally, for the **Hash meal**, we served pre-cooked diced mixed vegetables and chicken bits (ratio 2:1; Findus AB, Sweden), identical to our usual testing food in the past (references [3,16,30] of the main text). Similarly, in BD, we served an identical **Hash meal** twice to each participant and a **Meatballs** and potatoes **meal**, prepared with blended pork and beef minced meat (20% fat), salt and pepper, potatoes boiled for 30 minutes and served with a tomato based sauce (Mars Inc., USA). All the foods were precooked, refrigerated and re-heated just before serving, with the exception of the Porridge meal which was cooked at a nearby kitchen on the spot. The researchers took care to allow the foods to cool down to a comfortable temperature before presenting them to the study participants.
